# Cell Fate Determination of Lymphatic Endothelial Cells

**DOI:** 10.3390/ijms21134790

**Published:** 2020-07-06

**Authors:** Young Jae Lee

**Affiliations:** 1 Department of Biochemistry, College of Medicine, Gachon University, Incheon 21999, Korea; leeyj@gachon.ac.kr; Tel.: +82-32-899-6590; 2 Lee Gil Ya Cancer and Diabetes Institute, Gachon University, Incheon 21999, Korea

**Keywords:** lymphangiogenesis, lymphatic vessels, lymphatic endothelial cells, cell fate, genetically modified mice

## Abstract

The lymphatic vasculature, along with the blood vasculature, is a vascular system in our body that plays important functions in fluid homeostasis, dietary fat uptake, and immune responses. Defects in the lymphatic system are associated with various diseases such as lymphedema, atherosclerosis, fibrosis, obesity, and inflammation. The first step in lymphangiogenesis is determining the cell fate of lymphatic endothelial cells. Several genes involved in this commitment step have been identified using animal models, including genetically modified mice. This review provides an overview of these genes in the mammalian system and related human diseases.

## 1. Introduction

The lymphatic vascular system is essential for fluid homeostasis, dietary fat uptake, and immune responses [1,2,3]. The lymphatic vasculature is a one-way drainage system that transports lymph collected from the tissues to the venous vascular system. Defects in the lymphatic vascular system are associated with various types of human diseases such as lymphedema, obesity, atherosclerosis, inflammation, and fibrosis [1,2,3]. The lymphatic vasculature develops at embryonic day (E) 9.5 in mice and at the end of the 5^th^ week of gestation in humans, which occurs after the establishment of a primitive cardiovascular system [4,5,6]. In mice, lymphatic endothelial cells derived from the cardinal and intersomitic veins sprout to form the primitive lymphatic sac [6,7,8,9,10]. The primary lymphatic plexus produced by the proliferation of lymphatic endothelial cells in the lymphatic sac is remodeled and matured into the functional lymphatic vasculature [7,8,9,10,11,12]. In addition to venous endothelial cells, non-venous endothelial cells contribute to the formation of the lymphatic vasculature in various organs [13,14,15,16]. Many genes are involved in the development of the lymphatic vascular system. Phenotypic analyses of knockout mouse strains of these genes and lineage tracing experiments using reporter mouse strains provide valuable information to this field.

Most lymphatic endothelial cells are derived from venous endothelial cells [6]. Therefore, it is an important question as to how venous endothelial cells are committed to the lymphatic endothelial cell fate. This review focuses on the genes involved in the cell fate determination of lymphatic endothelial cells of mouse embryos. Mutations in these genes are associated with several human congenital and adult-onset diseases.

## 2. Origins of Lymphatic Endothelial Cells

The origin of lymphatic endothelial cells has been debated for more than 100 years. In 1902, Sabin proposed that the lymphatic sac was derived from venous endothelial cells based on an ink injection experiment using porcine embryos [17]. However, Huntington and McClure proposed in 1910 that mesoderm-derived endothelial precursor cells, independent of venous endothelial cells, formed the lymphatic sac and then connected to the venous vascular system [18]. Lineage tracing experiments using genetically modified mice by Srinivasan et al. showed that most lymphatic endothelial cells were derived from the cardinal and intersomitic veins, which strongly supported Sabin’s theory [6]. There is no doubt that veins are the main source of lymphatic endothelial cells [6]. However, recent studies have identified other progenitor cells that contribute to the formation of lymphatic vessels in specific tissues. During the development of mouse mesenteric lymphatics, mesenteric lymphatic endothelial cells are derived from *cKit*^+^-hemogenic endothelium-derived cells as well as venous endothelium-derived cells [14]. This dual source mechanism is observed in other tissues using lineage-tracing experiments in mice. Lymphatic endothelial cells in the cervical and thoracic skin originate from the venous-derived lymphatic sac, while some lymphatic vessels in the lumbar region are produced by vasculogenesis with non-venous endothelial cells [15]. Klotz et al. proposed that the cardiac lymphatic vessels were composed of lymphatic endothelial cells with heterogeneous cellular origins, venous- and non-venous cells [16]. Hemogenic endothelial cells in the yolk sac were suggested as the origin of the non-venous cells [16].

## 3. Specification of Lymphatic Endothelial Cells

### 3.1. Transcription Factor Prospero Homeobox 1 (PROX1)

PROX1 is a homeobox-containing transcription factor and the mammalian homolog of the *Drosophila prospero* gene with the consensus binding motif, C(A/T)(C/T)NNC(T/C) [19,20]. This gene is the master switch that determines the fate of lymphatic endothelial cells and also maintains their identity [4,7,21,22,23,24,25,26]. In mice, the biased expression of *Prox1* in endothelial cells of the cardinal vein in the jugular region specifies a subset of venous endothelial cells as lymphatic endothelial progenitor cells at around E9.5 [6,7,21]. *Prox1*^−/−^ embryos die around E14.5 and lack lymphatic vessels [7]. Loss of *Prox1* at early developmental stages (in the venous lymphatic endothelial progenitors) causes scattered blood-filled lymphatic vessels and cutaneous edema [22]. Overexpression of *Prox1* in endothelial cells leads to dermal edema and anemia at E14.5 and reprogramming of the identity of venous endothelial cells [26]. In addition to these in vivo experiments, ectopic overexpression or knockdown of *PROX1* in blood vascular endothelial cells or lymphatic endothelial cells disturbs the expression of lymphatic endothelial cell markers and blood vascular endothelial cell markers in these cells. Ectopic expression of *PROX1* in primary human dermal microvascular endothelial cells increases the expression of many lymphatic endothelial cell markers such as *PDPN* and *FLT4*/*VEGFR3* [23,27]. Ectopic expression *PROX1* also decreases the expression of many blood vascular endothelial cell markers, such as *NRP1*, *ICAM1*, *STAT6*, and *AXL* [23,27]. Knockdown of *PROX1* expression by siRNA in primary human lymphatic endothelial cells results in the downregulation of lymphatic endothelial cell markers, *PDPN* and *CCL21*/*SLC*, and in the ectopic expression of blood vascular endothelial cell markers, such as *ENG* and *CD34* [22]. These in vitro and in vivo data demonstrate that PROX1 is necessary and sufficient for the cell fate determination of lymphatic endothelial cells. PROX1 expression is regulated by several transcription regulators, including SRY-Box Transcription Factor 18 (SOX18) [28], Nuclear Receptor Subfamily 2 Group F Member 2 (NR2F2/COUP-TFII) [6], Hematopoietically Expressed Homeobox (HHEX) [29], Yes-Associated Protein 1 (YAP1) [30], and Tafazzin (TAZ) [30].

### 3.2. Transcriptional Regulators of PROX1

The transcription factor SOX18 is a member of the SOX (SRY-related HMG-box) family and has the consensus binding motif AACAAAG [31]. SOX18 binds directly to the *Prox1* promoter and activates its transcription [28]. *Sox18*^−/−^ mice die around E14.5 with a complete blockade of the differentiation of lymphatic endothelial cells from endothelial cells in the cardinal vein [28]. Overexpression of *Sox18* in blood vascular endothelial cells induces expression of lymphatic endothelial cell markers such as *Prox1*, *Efnb2*, and *Flt4*/*Vegfr3* [28]. The RAS-RAF1-MEK-ERK signaling cascade induces *SOX18* expression, and thus this signaling is important for the cell fate determination of lymphatic endothelial cells [32,33]. Endothelial cell-specific expression of human RAF1 S259A mutant (*RAF1*^S259A^), which induces constitutive activation of ERK, causes embryonic lethality at E15.5, enlarged lymphatic sacs and vessels, subcutaneous edema, cardiac defects, and induction of *Sox18* and *Prox1* expression [32]. SOX18 is necessary for *Prox1* expression, although on its own it is not sufficient [34]. NR2F2, an orphan nuclear receptor transcription factor, is required to activate *Prox1* expression in the cardinal vein by direct binding to the *Prox1* promoter [6,34]. *Nr2f2*^−/−^ mice die before E11.5 with defects in heart development and angiogenesis including malformations in the cardinal vein [35]. Endothelial cell-specific disruption of *Nr2f2* using *Tek*-cre causes ectopic expression of arterial markers in the veins and reduction of the number of *Prox1*^+^ cells in and around the cardinal vein [6,34,36]. NR2F2 specifies the fate of lymphatic endothelial cells by physically interacting with PROX1 in the lymphatic endothelial cells [24,37]. Recent studies have identified other transcriptional regulators of *PROX1*. HHEX is a member of the homeobox family of transcription factors and is expressed in endothelial cells of the cardinal vein [29]. Embryonic lethality caused by disruption of *Hhex* begins around E11.5 showing growth retardation, pericardial edema, vascular patterning defects, blood-filled lymphatic vessels, and a reduced number of *Prox1*^+^ cells within the cardinal vein [29,38]. Similar phenotypes are also observed in *Hhex*^flox/flox^;*Tek*-cre embryos [29]. Disruption of *Hhex* from E10.5 using *Prox1*-CreER leads to lymphatic defects, such as edema, blood-filled lymphatic vessels, and shorter, wider, and fewer branched lymphatic vessels [29]. Blood vessels, however, are not affected in these *Hhex*^flox/flox^;*Prox1*-CreER embryos [29]. Chromatin immunoprecipitation analysis indicates the direct binding of HHEX in the *Prox1* promoter [29]. YAP1 and TAZ are downstream effectors of the Hippo signaling pathway [39]. They translocate into the nucleus where they bind to TEAD/TEF transcription factors and function as transcriptional co-regulators [39]. In the cardinal vein, YAP1 and TAZ are in the cytoplasm of most *Prox1*^+^ lymphatic endothelial cells, whereas in blood vascular endothelial cells, YAP1 can be found in the nucleus and TAZ in the nucleocytoplasm [30]. Hyperactivation of YAP1 and TAZ in *Prox1*^+^ lymphatic endothelial progenitors results in a reduced number of *Prox1*^+^ lymphatic endothelial cells and decreased width of lymphatic sac [30]. Furthermore, hyperactivation of YAP1 and TAZ in *Cdh5*^+^ whole endothelial cells, including lymphatic endothelial progenitors, shows similar defects [30]. Hyperactivation of YAP1 in primary cultured human dermal lymphatic endothelial cells leads to the dedifferentiation of lymphatic endothelial cells to blood vascular endothelial cells [30]. In human dermal lymphatic endothelial cells, YAP1 and TAZ negatively regulate *PROX1* expression [30]. YAP1 may directly inhibit *PROX1* transcription through the recruitment of the NuRD complex and TEAD-mediated binding to the *PROX1* promoter [30].

### 3.3. Post-Transcriptional Regulators of PROX1 and Post-Translational Modification for PROX1

MicroRNAs (miRNAs), which are non-coding RNAs, are involved in the regulation of PROX1 expression [40,41]. *Mir181a* binds directly to the 3′-untranslated region of *Prox1*, causing degradation of *Prox1* transcripts and inhibition of *Prox1* translation [40]. Ectopic expression of *Mir181a* in primary lymphatic endothelial cells leads to reduced *Prox1* mRNA and protein levels and reprogramming of lymphatic endothelial cells to endothelial cells with blood vascular endothelial cell identity [40]. Conversely, knockdown of endogenous *Mir181a* in primary blood vascular endothelial cells increases *Prox1* expression [40]. Another miRNA, *MIR31*, which is identified as a blood vascular endothelial cell-specific miRNA, inhibits the translation of *PROX1* [41]. Post-translational modifications enable the functional diversity of the target protein. PROX1 is a target for small ubiquitin-like modifier 1 (SUMO1), and inhibition of the PROX1 sumoylation reduces the DNA binding and transcriptional activities of PROX1 [42].

### 3.4. FMS-Like Tyrosine Kinase 4 (FLT4)/Vascular Endothelial Growth Factor Receptor 3 (VEGFR3) Signaling

FLT4, also known as VEGFR3, is a member of receptor tyrosine kinases and is a receptor of the lymphangiogenic growth factor Vascular Endothelial Growth Factor C (VEGFC) that induces the budding-off of lymphatic endothelial cells from the cardinal vein [12]. *Vegfc*^−/−^ embryos die after E15.5 and show edema [12]. In *Vegfc*^−/−^ embryos, *Prox1*^+^ lymphatic endothelial cells fail to bud from the cardinal vein and remain trapped in veins [10,12]. The number of lymphatic endothelial progenitor cells in the cardinal vein is reduced in *Vegfc*^−/−^ embryos [25]. FLT4 is expressed in blood vascular endothelial cells until around E10.5, and its deficiency results in embryonic death after E10.0, severe cardiovascular defects, yolk sac vasculature defects, pericardial edema, and growth retardation [43]. Moreover, its expression in blood vascular endothelial cells is decreased, and in lymphatic endothelial cells, it is increased during lymphangiogenesis [21,43,44]. *Flt4* is a direct transcriptional target of PROX1 [25]. FLT4 signaling is required to maintain *Prox1* expression in lymphatic endothelial progenitor cells, which maintain the identity of lymphatic endothelial progenitor cells [25]. Ligand binding induces autophosphorylation of FLT4, which leads to the activation of downstream signaling pathways involved in the growth and survival of blood vascular endothelial cells and lymphatic endothelial cells [45,46]. The interaction between β1 integrin (ITGB1) and FLT4 is vital for the activation of FLT4 signaling [47,48,49]. A recent study has shown that integrin–linked kinase (ILK), a mechanosensitive regulator of FLT4, interferes with the interaction between β1 integrin and FLT4 [50]. The inhibition of *MIR126* in human lymphatic endothelial cells leads to the downregulation of *KDR*/*VEGFR2* and *FLT4*, as well as an inadequate response to VEGFA and VEGFC [51]. Two *Mir126*^−/−^ mouse strains with different genetic backgrounds show distinct embryonic phenotypes [51,52]. One of them shows partial embryonic lethality, edema, hemorrhage, and growth retardation [52]. Although the other is generally normal, loss of *Mir126* in *Flt4*^+/−^ causes embryonic lethality and severe edema [51].

### 3.5. NOTCH Signaling

NOTCH signaling is an evolutionary conserved pathway and is important for various biological processes such as cell fate determination, proliferation, differentiation, and homeostasis in both embryonic and adult stages. NOTCH signaling is essential for the tip/stalk cell selection and arterial specification during angiogenesis [53,54]. Ligand binding induces two sequential proteolytic cleavages in NOTCH and results in the release of NOTCH intracellular domain (NICD) from the membrane [55]. NICD translocates into the nucleus and interacts with recombination signal binding protein for immunoglobulin kappa J region (RBPJ) to regulate transcription of downstream targets [55]. NOTCH signaling is also involved in the cell fate determination of lymphatic endothelial cells and their cellular activities. NOTCH and NR2F2 mutually inhibit their expression [36,56,57]. In human dermal lymphatic endothelial cells, NOTCH downregulates *PROX1* and *NR2F2* expression through Hairy/enhancer-of-split related with YRPW motif 1 (HEY1) and HEY2, NOTCH-downstream transcription factors, whereas PROX1 and NR2F2 attenuate the FLT4 signaling that suppresses NOTCH signaling [56]. Chen et al. have shown that NR2F2 has a direct and negative regulatory effect on the expression of Neuropilin 1 (NRP1) and Forkhead box C1 (FOXC1), which are upstream activators of the NOTCH signaling [57]. In the cardinal vein of E9.75 mouse embryos, the NOTCH1 expressed region is on the opposite side of the PROX1 expressed region [58]. At E10.5, NOTCH1 and PROX1 show distinct and overlapping expression patterns in the posterior cardinal vein [58]. Disruption of *Notch1* in *Prox1*^+^ cells at E9.75 leads to mild edema, bold-filled lymphatic vessels, and enlarged lymphatic sac in E14.5 embryos [58]. The mutant embryos have an increased number of *Prox1*^+^ cells within the cardinal vein, as well as an increased number of *Prox1*^+^ cells emerging from the cardinal vein due to defects in the cell fate determination of lymphatic endothelial cells [58]. They have lymphatic vessels that are not correctly connected to the cardinal vein, causing blood-filled lymphatic vessels [58]. Another group has reported enlarged lymphatic vessels, and increased proliferation and survival of lymphatic endothelial cells in mutant embryos, in which *Notch1* is disrupted in *Prox1*^+^ cells at E10.5 [59]. In contrast, the expression of constitutively active NOTCH1 in *Prox1*^+^ cells downregulates the expression of *Prox1* and lymphatic endothelial cell markers [58]. Ectopic expression of constitutively active NOTCH1 in *Prox1*^+^ cells at E10.5 forms numerous small and disorganized lymphatic sac-like structures beside the cardinal vein, instead of at the jugular lymphatic sac [58]. Laminar flow-induced shear stress reduces NOTCH1 activity in lymphatic endothelial cells [60].

### 3.6. Bone Morphogenetic Protein (BMP) Signaling

Bone morphogenetic proteins (BMPs) are members of the transforming growth factor-β (TGF-β) superfamily. In the canonical BMP signaling pathway, the BMP ligand–receptor complex phosphorylates receptor-regulated SMADs (R-SMADs) by the Ser/Thr kinase activity of activated type I receptors [61]. Activated R-SMADs translocate into the nucleus with the common SMAD (SMAD4) and regulate downstream targets [61]. An experiment using BMP response element (BRE)-reporter mice shows that BMP-SMAD signaling is active in endothelial cells of the cardinal vein and lymphatic endothelial cells budding from the cardinal vein [61]. BMP2-SMAD signaling negatively regulates *PROX1* expression through induction of *MIR181a* and *MIR31* expression [62]. *Bmp9*^−/−^ embryos and neonates show enlarged lymphatic vessels and defective lymphatic valve formation [63,64]. In primary cultured human dermal lymphatic endothelial cells, BMP9 treatment directly downregulates *PROX1* expression through ACVRL1, a TGF-β type I receptor, and reduces the number of lymphatic endothelial cells [64].

### 3.7. Transmembrane Protein 100 (TMEM100)

*TMEM100* is identified as a downstream target of the BMP9/10-ACVRL1 pathway by my and other groups [65,66,67]. Expression of TMEM100 is highly induced by BMP9 treatment in the human umbilical artery and vein endothelial cells [66,68]; it is reduced in *Acvrl1*-deficient embryos and adults [65,66,67]. *Tmem100*^−/−^ embryos die between E10.5 and E11.5 with severe cardiovascular defects due to downregulated NOTCH and AKT signaling [65,66,67]. Recently, we have revealed that TMEM100 is essential for the cell fate determination of lymphatic endothelial cells by regulating NOTCH signaling [69]. Deletion of *Tmem100* in whole embryos at E10.5 leads to mild edema, blood-filled lymphatic vessels, lymphatic vessel dilation, and an increased number of *Prox1*^+^ lymphatic endothelial cells in the cardinal vein [69]. These defects are associated with a decreased NOTCH activity in endothelial cells of the cardinal vein [69]. Overexpression of TMEM100 in *Tek*^+^ endothelial cells results in embryonic lethality around E15.5, severe lymphedema, and small and disorganized lymphatic vessels [69]. In these overexpression embryos, the number of *Hey2*^+^ endothelial cells is increased in the cardinal vein, which is the exact opposite phenotype of *Tmem100*-deficient embryos [69].

## 4. Human Diseases Associated with Genes for the Cell Fate Determination of Lymphatic Endothelial Cells

Abnormal lymphangiogenesis is the cause of several congenital human diseases. The null mutations of genes that are highlighted in this review may cause human embryonic death. However, heterozygous mutations, missense mutations, or single-nucleotide polymorphisms (SNPs) in these genes can lead to human diseases. Hypotrichosis-lymphedema-telangiectasia syndrome (HLTS, OMIM #607823) and hypotrichosis-lymphedema-telangiectasia-renal defect syndrome (HLTRS, OMIM #137940) are caused by mutations in *SOX18*. HLTS is characterized by unusual associated symptoms, hypotrichosis, lymphedema, and telangiectasia [70]. HLTRS patients show renal defects as well as symptoms that overlap with HLTS [71]. Noonan syndrome 5 (NS5, OMIM #611553) and LEOPARD syndrome 2 (LPRD2, OMIM #611554) are caused by heterozygous mutations in *RAF1* [72,73,74,75,76,77,78]. Noonan and LEOPARD syndromes are caused by increased RAS signaling and show overlapping clinical features such as cardiac abnormalities, short stature, and facial dysmorphia [72,73]. Lymphatic dysplasia is also common in patients with Noonan syndrome [79,80]. Lymphatic malformation-1 (LMPHM1, OMIM #153100), also known as primary congenital lymphedema, is usually caused by heterozygous mutations in *FLT4*. In an LMPHM1 patient, Ghalamkarpour et al. reported a homozygous missense mutation (c.2563G.A; p.A855T) in *FLT4* [81]. Lymphatic malformation-4 (LMPHM4, OMIM #615907) is caused by heterozygous mutations in *VEGFC* [82]. Primary lymphedema is a chronic swelling of body parts due to malformations in the lymphatic system. Moreover, it has been elucidated that these diseases are caused by mutations in several genes including *GJC2*, *PIEZO1*, *EPHB4*, *CALCRL*, *FOXC2*, *SOX18*, *GATA2*, *CCBE1*, *PTPN14*, *KLF11*, and two genetic loci, as well as *VEGFC* and *FLT4* [83,84]. Most of the genes are upstream or downstream genes of PROX1-FLT4 signaling [84]. In addition to these human congenital diseases, *PROX1* mutation or SNPs are associated with adult-onset obesity or type 2 diabetes [85,86,87,88,89,90]. Although most *Prox1*^+/-^ pups die shortly after birth, some can survive to adulthood and show adult-onset obesity [85]. In humans, several studies have shown reduced *PROX1* expression in hyperlipidemia, obesity, and type 2 diabetes patients [86,87,88,89,90,91,92,93,94]. Genome-wide association studies have indicated that SNPs linked to the *PROX1* locus, such as rs1704198 and rs340874, are associated with these metabolic disorders [87,88,90,91,92,93,94].

## 5. Conclusions

**Figure 1:**
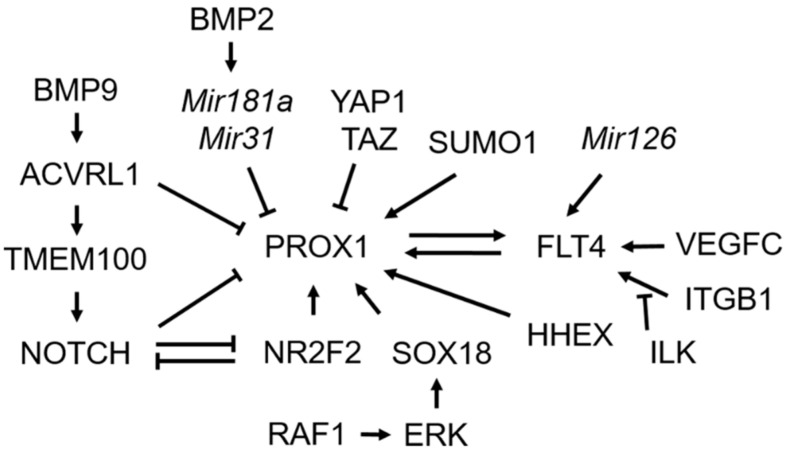
Genes and signaling pathways involved in the cell fate determination of lymphatic endothelial cells. PROX1 is the master regulator to determine the fate of lymphatic endothelial cells. Several genes act as transcriptional activators (*NR2F2*, *SOX18*, and *HHEX*) or repressors (*YAP1* and *TAZ*). RAF1/ERK signaling activates *SOX18* and *PROX1* expression. MiRNAs, *Mir181a* and *Mir31*, are post-transcriptional regulators of *PROX1*. The sumoylation of PROX1 by SUMO1 modulates the DNA binding and transcriptional activities of PROX1. BMP2 signaling negatively regulates *PROX1* expression through an increase of *Mir181a* and *Mir31* expression. NOTCH and NR2F2 mutually inhibit their expression, and NOTCH downregulates PROX1 and NR2F2 expression via HEY1 and HEY2. BMP9/ACVRL1 signaling inhibits *PROX1* expression. TMEM100, a downstream target of BMP9/ACVRL1 signaling, activates NOTCH signaling. VEGFC is a lymphangiogenic growth factor and the ligand of FLT4. VEGFC-FLT4 signaling that is a main downstream effector of PROX1 is essential for the budding-off of lymphatic endothelial cells from the cardinal vein. Downregulation of *Mir126* attenuates FLT4 signaling. Interaction between FLT4 and ITGB1, which is interfered with by ILK (integrin-linked kinase) is important for the activation of FLT4 signaling.

This review focuses on important genes and signaling pathways involved in the cell-fate determination of lymphatic endothelial cells, based on studies using genetically modified mice (Figure 1, Table 1). Although our knowledge of lymphangiogenesis has improved, there are still many points to be elucidated in disease conditions, even under normal development conditions. Since the function of PROX1 that determines the cell fate of lymphatic endothelial cells during early development has been elucidated, the functions of various genes related to PROX1-FLT4 signaling have been reported, and thus our understanding of this biological process has deepened. However, the identification of new genes such as *HHEX*, *YAP*, *TAZ*, *ILK*, *MIR126*, and *TMEM100*, which are involved in the cell fate determination of lymphatic endothelial cells, suggests that many important genes have not yet been identified in this field. If we better understand the cell fate determination of lymphatic endothelial cells during the development of lymphatic vessels in various organs as well as in early embryos, this would give us an opportunity for therapeutic intervention.

Studies using genetically modified animals, especially mice, have provided us with a great deal of information about lymphangiogenesis. The production of genetically modified mice was a time-consuming and labor-intensive task in the past. However, the recently developed CRISPR/Cas9 system can reduce these efforts. CRISPR/Cas9 can also enable the production of more precisely designed mice [95]. In the future, these mice will not only provide a better understanding of lymphangiogenesis but will also help find therapeutic solutions for related diseases.

## Figures and Tables

**Table 1:** Mouse models of genes involved in the cell fate determination of lymphatic endothelial cells.

Gene	Roles in Lymphangiogenesis	Viability and Gross Morphology of Knockout (KO) Embryos	Human Diseases ^1^
*Prox1*	Specification and maintenance of lymphatic endothelial cells	KO mice [7,21] die ~E14.5; lymphedema; lack of lymphatics cKO mice (*Tek*-cre) ^2^ [6] lymphedema; compromised lymphangiogenesis cKO mice (*CAGGCreER*, E8.5~E10.5, E12.5 and E13.5) ^3^ [22] lymphedema; blood-filled lymphatics OE mice (*tie1 tTA:tetOS prox1*) ^4^ [26] lymphedema; anemia	Human SNP rs1704198 located in the proximity of *PROX1* associated with a larger waist circumference Human SNP rs340874 located in the 5′-UTR of *PROX1* associated with fasting glycemia and type 2 diabetes
*Sox18*	Activation of *Prox1* expression	KO mice [28] die ~E14.5; lymphedema; lack of lymphatics	Hypotrichosis-lymphedema-telangiectasia syndrome (OMIM #607823) Hypotrichosis-lymphedema-telangiectasia-renal defect syndrome (OMIM #137940)
*Raf1*	Activation of *Sox18* and *Prox1* expression through ERK signaling	KO mice [96,97] die after E11.5 (until E16.5); growth retardation; defects in several organs including the skin, eyelids, lung, placenta, and liver OE mice (*VE-cadherin-tTA/RAF1^S259A^*) ^4^ [32] die at E15.5; lymphedema; enlarged lymphatics; heart defects; induction of *Sox18* and *Prox1* expression	Noonan syndrome 5 (OMIM #611553) LEOPARD syndrome 2 (OMIM #611554) Cardiomyopathy, dilated, 1NN (OMIM #615916)
*Nr2f2*	Activation of *Prox1* expression Inhibition of NOTCH signaling	KO mice [35] die before E11.5; heart defects; angiogenesis defects cKO mice (*Tek*-cre) [6,34,36] die at E11.5; compromised lymphangiogenesis; ectopic expression of *Notch1*	46, XX sex reversal 5 (OMIM #618901) Congenital heart defects, multiple types, 4 (OMIM #615779)
*Hhex*	Activation of *Prox1* expression	KO mice [29,38] die after E11.5; pericardial edema; blood-filled lymphatics; growth retardation; vascular patterning defects cKO mice (*Tek*-cre) [29] die after E11.5; pericardial edema; growth retardation; vascular patterning defects; blood-filled lymphatics; lymphedema; defects in lymphatic vessels cKO mice (*Prox1*-CreER, E10.5~E12.5) [29] blood-filled lymphatics; lymphedema; defects in lymphatic vessels	
*Yap1* and *Taz*	Inhibition of *Prox1* expression	Double cKO mice (*Prox1*-CreER, E11.5 and E13.5) [30] lymphedema; defects in lymphatic vessels	YAP1: Coloboma, ocular, with or without hearing impairment, cleft lip/palate, and/or mental retardation (OMIM #120433) TAZ: Barth syndrome (OMIM #302060)
*Vegfc*	Ligand for FLT4 Budding-off of lymphatic endothelial cells	KO mice [10,12,25] die after E15.5; lymphedema; failure of the budding-off of lymphatic endothelial cells from the cardinal vein	Lymphatic malformation 4 (OMIM #615907)
*Flt4*	Receptor for VEGFC Budding-off of lymphatic endothelial cells	KO mice [43] die after E10.5; severe cardiovascular defects; yolk sac vasculature defects; pericardial edema; growth retardation	Lymphatic malformation 1 (OMIM #153100) Congenital heart defects, multiple types, 7 (OMIM #618780) Hemangioma, capillary infantile, somatic (OMIM #602089)
*Ilk*	Inhibition of the interaction between β1 integrin and FLT4	cKO mice (*Kdr*-cre) [50] die after E13.5; lymphedema; head bleeding; enlarged lymphatics; lymphatic and blood vascular sprouting defects	
*Mir126*	Control of FLT4 signaling	KO mice 1 [51] No obvious defects KO mice 2 [52] partial embryonic lethality; edema; hemorrhage; growth retardation *Mir126^-/-^; Flt4^+/-^* [51] Die before birth; lymphedema at E14.5	
*Notch1*	Inhibition of *Prox1* and *Nr2f2* expression	cKO mice (*Prox1*-CreER, E9.75) [58] mild lymphedema; bold-filled lymphatics; enlarged lymphatic sacs cKO mice (*Prox1*-CreER, E10.5) [59] enlarged lymphatic vessels OE mice (*Prox1*-CreER, E10.5) [58] numerous small and disorganized lymphatic sac-like structures	Adams-Oliver syndrome 5 (OMIM #616028) Aortic valve disease 1 (OMIM #109730)
*Bmp9*	Downregulation of *Prox1* expression through ACVRL1	KO mice [63,64] enlarged lymphatic vessels; defective lymphatic valve formation	Telangiectasia, hereditary hemorrhagic, type 5 (OMIM #615506)
*Tmem100*	Inhibition of NOTCH signaling	cKO mice (ROSA26-CreER, E10.5) [69] die around E16.5; lymphedema, blood-filled lymphatic vessels; lymphatic vessel dilation OE mice (*Tek*-cre) [69] die around E15.5; lymphedema, small size and number of lymphatic vessels	

^1^ Human diseases associated with each gene are listed with OMIM number [83]. ^2^ The cre mouse strains that are used for Cre/loxP recombination in conditional knockout (cKO) or overexpression (OE) mice are listed in the parentheses. ^3^ Tamoxifen is treated at indicated embryonic day(s) for inducible Cre-loxP recombination in cKO or OE mice. ^4^ Overexpression mice using doxycycline-induced Tet-off system.

## Abbreviations

BMP	Bone morphogenetic protein
E	Embryonic day
HLTRS	Hypotrichosis-lymphedema-telangiectasia-renal defect syndrome
HLTS	Hypotrichosis-lymphedema-telangiectasia syndrome
LMPHM1	Lymphatic malformation-1
LMPHM4	Lymphatic malformation-4
MiRNA	MicroRNA
NICD	NOTCH intracellular domain
R-SMAD	Receptor-regulated SMAD
*RAF1* ^S259A^	RAF1 S259A mutant
SNP	Single-nucleotide polymorphism
TGF-β	Transforming growth factor-β
